# Notes and News

**Published:** 1893-01-28

**Authors:** 


### Notes and News.

Collected and Communicated.

The German Hospital, Dalston, was the scene of a fire last week. Fortunately it originated in the out-patients' department, and owing to prompt measures did not spread beyond the waiting-room, which was severely damaged. It is believed that the conflagration was caused by a spark from the grate alighting on the boards of the flooring.

The straightforward explanation given by Dr. Davies, in reply to the paragraph headed "Guy's Hospital Again: Allegations Against a Doctor," in the *Morning*, should really shame that portion of the press which lends its columns so readily to sensational paragraphs which constantly prove most unwarranted, and which, however completely explained away, are apt to leave a stain on the fame of institutions which only exist for the public weal.

Some important decisions were arrived at when the Metropolitan Asylums Board held their last meeting on Saturday. It was determined that the Local Government Board is to be asked to assent to the permanent appropriation of the site of the North-Eastern Hospital at Tottenham for the purposes of a fever hospital, and that the temporary structures which have been erected during the past year should be allowed to remain standing until the permanent buildings are completed. The third item on the programme, which was the proposal to purchase the Tooting Lodge estate for a sum of £28,000, for the purposes of an acute hospital, provoked opposition on the grounds of expense and the harm which would possibly accrue to a popular neighbourhood. All three propositions are, however, to be submitted to the Local Government Board for decision. The present permanent fever hospitals of the Metropolitan Asylums Board accommodate but 2,292 patients, whilst during the past year as many as 4,389 had to be received at one time. It is therefore proposed that the accommodation should be increased to at least 4,500 beds, which even then leaves a very small margin for increase of population.

The Hospital Saturday Fund has now awarded the £17,000 collected in 1892. Twenty-nine general hospitals have received £6,280 between them, 59 special hospitals £6,169, 33 dispensaries £1,054, 15 convalescent homes £1,482, 18 miscellaneous £1,913, and £100 has been distributed in supplementary grants. Most of the general hospitals have received increased awards this year, and one, the Queen's Jubilee Hospital, has been added to their number.

A most interesting little ceremony was that of the unveiling of the Albert Victor Memorial bed at the National Children's Hospital in Dublin. The occasion chosen was the anniversary of the death of the late Prince, who but shortly before his death opened the ward named after him at the hospital. Thus the memorial chosen by the people of Dublin is a very appropriate one, and already over £500 has been collected for the endowment. An album containing the names of the subscribers is to be presented to the Prince and Princess of Wales, to whom it must be most gratifying to know how far from forgotten is their son.

For nearly three years the guardians of Belfast have permitted senior medical students to receive clinical instruction in the wards of the Union Infirmary. An extension in favour of a larger number of students is now suggested, together with the granting of certificates and the increase of the medical staff to facilitate teaching. These suggestions have attracted the attention of those apparently unacquainted with the original custom, and now opposition to the admission of any students to the Infirmary has been raised. The objection seems to be one principally of sentiment. It is stated that the pauper patients will not like it. It is not possible to regard this argument in connection with the medical schools at our voluntary hospitals, and considering the ultimate advantage which will accrue to the many through the extended experience afforded we trust the malcontents will not carry the day.

Some important alterations have been made in the rules of two of the medical charities of Birmingham. The Queen's Hospital, finding the abuse of the out-patient department to be very serious, has decided to charge a fee of one shilling after patients have attended one consultation. Such a step as this is liable to throw more work upon the free hospitals, and will probably render it necessary that all should protect themselves in some manner or other. Many foreign countries are ahead of us in wise regulations as to out-patients. The patients furnish attested particulars of their circumstances at the outset, and the charges are made accordingly. The trouble of investigation is thus minimised, and the really indigent stand a fair chance. Whilst the Queen's has made a regulation to reduce expenditure, the General Hospital is about to increase its burden, but the additional expenses to be incurred is fully justified. The patients hitherto have been required to provide crockery, tea, sugar, and linen for themselves. This has led to abuses, principally in the direction of food introduced by these means into the hospital. Such regulations weigh heavily upon the very poor, and the abolition of them will, we feel sure, meet with universal approval. Metropolitan hospitals are not entirely free from regulations of the kind, and in institutions where they exist money would be well bestowed to render their abolition possible.

